# Integrating ethics into infectious disease modelling: case studies and perspectives from the COVID-19 pandemic

**DOI:** 10.1098/rsfs.2025.0046

**Published:** 2025-09-26

**Authors:** Cameron Zachreson, Joel C. Miller, Julian Savulescu

**Affiliations:** ^1^ School of Computing and Information Systems, The University of Melbourne, Parkville, Victoria, Australia; ^2^ Department of Mathematical and Physical Sciences, La Trobe University, Melbourne, Victoria, Australia; ^3^ Centre for Biomedical Ethics, Yong Loo Lin School of Medicine, National University of Singapore, Republic of Singapore; ^4^ Oxford Uehiro Institute, University of Oxford, Oxford, UK

**Keywords:** infectious disease, modelling, COVID-19, ethics, public health, public health ethics

This theme issue of *Interface Focus* presents a selection of research articles, perspectives and one targeted literature review on emerging topics within an exciting new interdisciplinary domain combining infectious disease modelling and public health ethics.

Mathematical and computational modelling has taken on a prominent role in helping decision makers choose how to respond to the public health threat posed by infectious diseases. The trend has been most visible in the context of COVID-19, which saw computational simulation approaches established as ubiquitous tools for the design of public health interventions around the world. While the usefulness of modelling is evident, we are concerned about the persistent disconnect from ethical principles in the flurry of technical development producing and applying state-of-the-art methods for modelling infectious disease interventions [1]. In scenarios such as pandemics, the laws, principles and norms safeguarding the rights and wellbeing of individuals can be justifiably relaxed to prevent widespread disease and preventable death. As such, the principles of public health ethics should be applied at all stages of the decision-making process, including modelling.

In public health ethics, the Four Principles of Beauchamp and Childress are foundational concepts: public health must respect autonomy, beneficence, non-maleficence and justice [2]. The enclosed contributions include case studies demonstrating how models can inform the application of the four principles and analyse trade-offs between them—for example, by optimizing objective functions that include the values of utility (aggregate health benefit) and equity (fair distribution of benefits and burdens, as an instantiation of justice). Together, these case studies illustrate the potential to expand modelling approaches to encompass a broader range of ethical considerations, including moral responsibility, duties of care towards dependants and social values beyond direct health benefit.

The principle of equity emerged as a motivating theme for much of this theme issue with several enclosed articles discussing it at length. These studies help examine the connection between theoretical challenges faced when modelling disease spread over heterogeneous populations and when designing equitable responses to disease outbreaks. When models focus solely on reducing aggregate disease burden, they risk obscuring how epidemics and interventions may unequally affect different populations. The contributions in this issue highlight these challenges and refine our understanding of the practical and ethical trade-offs that may arise due to heterogeneity in disease impacts, transmission mechanisms and intervention effects across population groups.

Trade-offs can exist between utility and equity, in the sense that interventions or policies supporting distributive justice may produce outcomes with lower aggregate health benefit across the population [3,4]. Understanding whether and how such trade-offs manifest is a major challenge, and the articles in this issue provide nuanced insights into it. The targeted review by Rumpler & Lipsitch highlights the implicit disregard for heterogeneity in almost all modelling studies focused on the problem of optimal vaccine allocation against COVID-19. Their paper then delves into the findings of the few studies that have examined the potential for a trade-off between equity and utility. For the case of COVID-19, the evidence they review refutes the idea that there must exist a trade-off between outcome equity and aggregate health benefit. The modelling study by Zarebski *et al.* further elucidates the mechanism of this finding, concluding that, for the case of vaccination against COVID-19, such trade-offs exist only for ethical edge cases. However, by expanding their analysis beyond equity in disease burden both Zarebski *et al.* and Young *et al.* identify cases where ethical and practical trade-offs appear robustly across a range of scenarios.

From a methodological point of view, the large-scale agent-based modelling study by Harris *et al.* provides a convincing demonstration of the insights gained from incorporating equity considerations into computational modelling of disease transmission. For instance, their work illustrates how accounting for correlations between social clustering mechanisms such as workforce composition, household size and school classroom size enables hypothesis tests aimed at understanding the driving causes of disparities in disease prevalence that are frequently observed between subpopulations with different characteristics such as ethnicity or race. We expect such findings to have major implications for the design of mitigation measures that aim to account for existing health disparities.

While the aftermath of the COVID-19 pandemic has emphasized the need to address questions of equitable public health policy, equity is only one facet of public health ethics. The broad scope of principles that should be considered in modelling studies is described by Silva *et al.*, using the illustrative example of school closures during COVID-19. Their perspective highlights the connection between the ability of models to account for unintended consequences beyond disease burden and the ability to carry out modelling studies in a way that supports justice and solidarity. The case study of COVID-19 school closures in Hong Kong described by Young *et al.* further illustrates this concept by demonstrating the dependency of optimal school closure policy on the weights given to different outcome measures including school days missed and screening costs. In showing this, Young *et al.* provide a detailed application of the general approach described by Zarebski *et al.*, which demonstrates that objective functions and optimization algorithms used for comparing potential policies can act as a conceptual bridge between mathematical models of infectious disease interventions and frameworks of public health ethics.

In such frameworks, autonomy is a central ethical value, often in tension with the imperative to mitigate disease transmission. This raises a key question: how can models quantify the loss of human agency introduced by public health interventions if they are unable to represent behavioural choice? Chang *et al.* take on the long-standing challenge of simulating human autonomy and endogenous behavioural responses to the risk of infection from a novel virus such as SARS-CoV-2. Their findings highlight the critical role of individual behaviour and risk perception in shaping epidemic dynamics, and they lay important groundwork for modelling the interplay between policy measures and self-organized risk avoidance. Models capable of simulating individual decision making in the context of a pandemic represent a foundational step towards incorporating autonomy into infectious disease modelling as a value that may trade off with aggregate health benefit.

Beyond the necessary theoretical and technical advancements, achieving a positive role of modelling in the design of ethical interventions will require us to approach the operational ethics of disease modelling as a social and scientific process. Silva *et al.* highlight how operational ethics in modelling demands attention not only to the outcomes our models inform but also to the modelling process: the stakeholders involved, how modellers engage with them and transparency during model development and analysis. To realize this vision, those of us in the modelling community must learn from our experiences while we navigate the shift of resources away from modelling capacity in the wake of COVID-19. Governments, universities and public health agencies must strategically invest in a sustainable modelling community with the resources and institutional support to engage ethically and efficiently in the design of public health interventions. We conclude the issue with a group perspective by Sherratt *et al.*, who reflect on the system-level challenges facing the modelling community as we consolidate the lessons learned from COVID-19 and prepare for the next pandemic.

To conclude, infectious disease modelling has become a ubiquitous and valuable tool for designing effective public health intervention policy. However, interventions informed by modelling can produce ethically contestable outcomes, such as COVID-19 lockdown policies that disproportionately burdened disadvantaged groups. Going forward, the modelling and ethics communities must work together to embed ethical considerations into state-of-the-art approaches to intervention design and implementation. Doing so promises not only to enhance the decision-making process but also to strengthen public engagement, promote social cohesion and support a shared commitment to the public health response [5].

## Data Availability

This article has no additional data.
